# Effective Knockdown of Gene Expression in Primary Microglia With siRNA and Magnetic Nanoparticles Without Cell Death or Inflammation

**DOI:** 10.3389/fncel.2018.00313

**Published:** 2018-09-21

**Authors:** Alejandro Carrillo-Jimenez, Mar Puigdellívol, Anna Vilalta, Jose Luis Venero, Guy Charles Brown, Peter StGeorge-Hyslop, Miguel Angel Burguillos

**Affiliations:** ^1^Departamento de Bioquímica y Biología Molecular, Facultad de Farmacia, Universidad de Sevilla, Seville, Spain; ^2^Instituto de Biomedicina de Sevilla (IBiS), Hospital Universitario Virgen del Rocío, CSIC, Universidad de Sevilla, Seville, Spain; ^3^Department of Biochemistry, University of Cambridge, Cambridge, United Kingdom; ^4^Department of Clinical Neurosciences, Cambridge Institute for Medical Research, University of Cambridge, Cambridge, United Kingdom

**Keywords:** microglia, siRNA, transfection, TREM2, CD33, CGC, Alzheimer’s disease

## Abstract

Microglia, the resident immune cells of the brain, have multiple functions in physiological and pathological conditions, including Alzheimer’s disease (AD). The use of primary microglial cell cultures has proved to be a valuable tool to study microglial biology under various conditions. However, more advanced transfection methodologies for primary cultured microglia are still needed, as current methodologies provide low transfection efficiency and induce cell death and/or inflammatory activation of the microglia. Here, we describe an easy, and effective method based on the Glial-Mag method (OZ Biosciences) using magnetic nanoparticles and a magnet to successfully transfect primary microglia cells with different small interfering RNAs (siRNAs). This method does not require specialist facilities or specific training and does not induce cell toxicity or inflammatory activation. We demonstrate that this protocol successfully decreases the expression of two key genes associated with AD, the triggering receptor expressed in myeloid cells 2 (TREM2) and CD33, in primary microglia cell cultures.

## Introduction

Microglia are the resident tissue macrophages of the central nervous system (CNS) where they survey for insults that may affect the homeostasis of the system (Kettenmann et al., 2011; Boche et al., 2013). They play several roles under physiological conditions, including synaptic pruning (Schafer et al., 2012; Um, 2017), but are also important during neurodegenerative diseases (Hirsch and Hunot, 2009; Rayaprolu et al., 2013; Nalls et al., 2014; Ulrich et al., 2014; Sims et al., 2017; Kang et al., 2018b). In the case for Alzheimer’s disease (AD), early studies in the 1990s showed the presence of activated immune cells in the brains of deceased people affected by AD (Eikelenboom et al., 1994), which suggested the hypothesis that the neuroinflammatory response plays a detrimental role during the disease progression. This hypothesis has been supported by many other reports, including some recent genome-wide association studies (GWAS) showing that several polymorphisms in innate immune genes are risk factors to develop AD (Rayaprolu et al., 2013; Mhatre et al., 2015; Sims et al., 2017).

Mechanistic studies in microglia cells heavily depends on *in vitro* approaches using different microglia cells lines (e.g., BV2, CHME3), induced pluripotent stem cell (iPS) derived cells or rodent primary microglia cell cultures. Cell lines are convenient because they do not require isolation and can be expanded indefinitely to provide high yields. However, during immortalization and repeated passaging, they may have acquired different features that are not present under physiological conditions in primary microglia cells (Butovsky et al., 2014). Working with iPS cells is also a very valuable tool due to their capabilities to be transformed into different cell types, including microglia cells. On the other hand, the process for expansion and transformation of iPS cells into microglia cells is a laborious and complicated procedure, with several different protocols to follow in the literature (Muffat et al., 2016; Brownjohn et al., 2018).

Hence, working with primary microglia cell cultures is important. However, working with primary microglia cell cultures presents challenges. One of the main limitations is the low yield produced from each animal and their limited survival time period in the absence of astrocytes. Also, primary microglia cell cultures are difficult cells to transfect, providing low efficiency of transfection and also are quite vulnerable to death when using traditional methods of transfection. One way to solve this problem has been to generate different transgenic mouse lines, as in the case of triggering receptor expressed in myeloid cells 2 (TREM2; Turnbull et al., 2006; Cheng et al., 2018; Filipello et al., 2018). Primary microglia cells are then isolated from these mice. However, this process is expensive and takes several months before you obtained the desired transgenic line. An alternative to generation of transgenic mice has been the use of transduction systems to overexpress or silence the expression of different protein targets. In particular, the use of lentiviral vectors has proven to be effective for this purpose (Masuda et al., 2013). However, the whole process can be challenging and requires the use of specific material (like class II security hoods) and special training for different type of tasks such as design of the virus’ sequence, choosing the right bacterial strain to avoid genomic rearrangements while amplifying the viral vector, stability of your viral stock to freeze and thaw cycles, efficiency of transduction depending on the concentration of your virus (virus tittering), or the usage of different reagents (for instance polybrene or fibronectin) to decrease the repulsive charges of the virus with the cell membranes to increase the transduction efficiency.

Here, we describe a simple method to knockdown the expression of different genes in primary microglia by using small interfering RNA (siRNA) and the Magnetofection™ principle patented by OZ Biosciences as a method of transfection. The Magnetofection™ method allow us to associate nucleic acids (in this case siRNA), with specific magnetic nanoparticles (made of iron oxide which is fully degradable). The resulting molecular complexes are then concentrated and transported into cells through an appropriate magnetic field. Therefore, the exploitation of a magnetic force exerted upon the siRNAs allows a very rapid concentration of the entire applied siRNA dose on cells, so that 100% of the cells get in contact with a significant vector dose and promotes cellular uptake. The cellular uptake of the genetic material is accomplished by endocytosis and pinocytosis, two natural biological processes. Consequently, membrane architecture and structure remain intact in contrast to other physical transfection methods that damage, create hole or electroshock the cell membranes.

To illustrate the use of this method in primary microglia we have knocked down the expression of TREM2 and CD33, two important genes whose mutations are considered a risk factor to develop AD (Griciuc et al., 2013; Colonna and Wang, 2016).

## Materials and Methods

### Reagents

LPS from *Salmonella enterica* serotype typhimurium (Sigma, catalog number L6511) was used for this study. Isolectin GS-IB4 from *Griffonia simplicifolia* (Alexa Fluor^®^ 568 conjugate) was purchased from Thermo Fisher (catalog number I21412). Glial-Mag kit was purchased from OZ Biosciences (catalog number KGL00250). The protocol used is based on the manufacturer’s recommendation, which we have optimized for a 24-well plate format (Supplementary Figure S1). The different siRNAs (positive—siGLO— and negative controls—non-targeting— and siTREM2 and siCD33) were purchased from Dharmacon (Horizon) and their sequences and catalogs numbers are provided in Table 1. A complete list of primers (ordered through Sigma Aldrich) with their sequences is provided in Table 2.

**Table 1:** Sequence and catalog numbers for the different small interfering RNAs (siRNAs).

siRNA	Catalog number	Sequence
siRNA non-targeting (1)	D-001810-10	UGGUUUACAUGUCGACUAA
siRNA non-targeting (2)	D-001810-10	UGGUUUACAUGUUGUGUGA
siRNA non-targeting (3)	D-001810-10	UGGUUUACAUGUUUUCUGA
siRNA non-targeting (4)	D-001810-10	UGGUUUACAUGUUUUCCUA
Mouse siCD33 (1)	J-047562-09	CAAUAAGAGACCCGGGACA
Mouse siCD33 (2)	J-047562-10	GCUCAAUGUUACCCGGAAA
Mouse siCD33 (3)	J-047562-11	GGAGCUUGCUGUUUAGGCA
Mouse siCD33 (4)	J-047562-12	GAGAACCUUUUGUGAGAUA
Mouse siTREM2 (1)	J-040918-09	CGGAGGUACGUGAGAGAAU
Mouse siTREM2 (2)	J-040918-10	GGUCAGAGGGCUGGACUGU
Mouse siTREM2 (3)	J-040918-11	CCUGCGUUCUCCUGAGCAA
Mouse siTREM2 (4)	J-040918-12	CUGAGUGGGAGGAGAACUA
Rat siTREM2 (1)	J-082332-09	CAGAAUGGGAGCACGGUCA
Rat siTREM2 (2)	J-082332-10	CGUCUGUACUUUGGACAUU
Rat siTREM2 (3)	J-082332-11	UAUCCCGGGAGCAGGAAUA
Rat siTREM2 (4)	J-082332-12	CCGAGGAGUCAGAGAGUUU

**Table 2:** Sequence for primers.

Name of gene	Forward sequence (5′→3′)	Reverse sequence (5′→3′)
Actb (mouse)	CCACACCCGCCACCAGTTCG	CCCATTCCCACCATCACACC
Actb (rat)	AAGACCTCTATGCCAACAC	TGATCTTCATGGTGCTAGG
Cd33 (mouse)	ATGAGAGAGCTGGTCCTGGT	CCCATGTGCACTGACAGCTT
Il1-β (mouse)	GTGCTGTCGGACCCATATGA	AGGCCACAGGTATTTTGTCGT
Nos2 (mouse)	CTGGGGCAGTGGAGAGATTT	TTGTCTCTGGGTCCTCTGGT
Tnfα (mouse)	GGTGCCTATGTCTCAGCCTC	ACTGATGAGAGGGAGGCCAT
Trem2 (mouse)	TCATCTCTTTTCTGCACTTC	TCATAAGTACATGACACCCTC
Trem2 (rat)	AAACAAGATCTGACACAAGG	CGTCATAAGTACATGACACC

### Animals

Two to five days-old wild-type mice (C57BL/6 background) and rats (Wistar) were obtained from Charles River Laboratories. All experiments were performed in accordance with the UK Animals (Scientific Procedures) Act (1986) and were approved by the Cambridge University local ethical committee.

### Generation of Primary Microglia Cultures From Postnatal Mouse and Rats

Primary microglia cultures were prepared from mice and rat pups at postnatal day P1–5 which were sacrificed by decapitation. Brains were removed in ice-cold Ca^2+^- and Mg^2+^-free Hanks Buffered Salt Solution (HBSS, Invitrogen) containing 10 μg/ml gentamicin (Sigma). Cortical hemispheres were dissected and meninges, blood vessels as well as white matter were removed. Tissue was cut into small pieces and transferred to pre-warmed HBSS containing 0.17% trypsin (37°C), chopped thoroughly and incubated for 15–20 min at 37°C. Supernatant was removed and the remaining trypsin was neutralized by addition of Dulbecco’s Modified Eagle’s Medium (DMEM, Invitrogen) supplemented with 10% fotal bovine serum (FBS) and gentamicin (10 μg/ml) in mice. In the case of rats, we added also 1–2 mg of Deoxyribonuclease from bovine pancreas (Sigma) in 25 ml of DMEM supplemented with 10% FBS and gentamicin (10 μg/ml). Tissue was mechanically dissociated by repeated trituration through a 10 ml and 5 ml sterile serological pipettes and finally through a 1 ml pipette tip (pipetted up and down 20 times with each one of the serological pipettes), leaving the suspension undisturbed for 1 min before collecting the supernatant. The supernatant was centrifuged at 150 *g* for 7 min at room temperature. We discarded the supernatant and resuspended the pellet in fresh media. The cell suspension was then filtered using first a 100 μm cell strainer and then a 40 μm cell strainer (BD Biosciences, San Jose, CA, USA). Both cell strainers were previously moistened with 2 ml of complete media to facilitate the filtering process. The cell suspension was centrifuged at 150 *g* for 7 min at room-temperature (RT). Supernatant was discarded, and the cell pellet was resuspended in DMEM supplemented with 10% FBS and 10 μg/ml gentamicin (Sigma) and seeded onto T75 flask (Nunc) coated with 0.0005% poly-L-lysine (Sigma) in PBS at a ratio of 3–4 pups (mouse) or 1 pup (rat) per T75 cell culture flask. After 24 h, the T75 flask was carefully tapped to dislodge sedimentary cell debris, and medium was exchanged (20 ml/flask). Cultures were maintained at 37°C in a humidified atmosphere of 5% CO_2_ and allowed to mature *in vitro* for 7–14 days before transfection. Microglial cells were harvested from confluent astrocyte monolayers, 14 days after the initial seeding, by a combination of tapping the side of the culture flask and gently vortexed for ~1-min supernatant containing detached microglial cells was collected and centrifuged at 150 *g* for 7 min at room temperature. These microglial cells found were plated into 24-well plates in conditioned media to a ratio 1:3 (media from flask: fresh media), coated with 0.0005% poly-L-lysine, at a density of 200,000 cells in 400 μl per well. Experiments were performed 48 h after the final plating.

### Primary Cerebellar Granule Cells (CGCs) Cultures From Mouse and Rat Pups

Primary mixed neuronal/glial cultures were prepared from cerebella of postnatal day 3–5 mice or rat pups as previously described (Kinsner et al., 2005). Briefly, pups were killed by decapitation and brains were quickly removed and placed in ice-cold HBSS containing 10 μg/ml gentamicin (Sigma). Then, the cerebella were separated from the brainstem, and meninges were removed. The tissue was transferred into pre-warmed Versene solution (37°C, Invitrogen), cut into small pieces and incubated for 5 min at 37°C, 5% CO_2_. Tissue was then mechanically dissociated using a sterile plastic pasteur pipettes, and then, with P1000 tips of decreasing aperture size. After each dissociation step, dissociated cells present in the Versene solution were added in pre-warmed DMEM supplemented with 5% horse serum, 5% FBS, 5 mM 4-(2-Hydroxyethyl) piperazine-1-ethanesulfonic acid (HEPES), 20 mM KCl, 2 mM L-glutamine, 13 mM glucose and 10 μg/ml gentamicin (all Sigma). Dissociated cells resuspended in supplemented DMEM were centrifuged at 150 *g* for 7 min at RT. The cell-suspension was passed through a 40 μm cell-strainer (BD Biosciences, San Jose, CA, USA) and cells were seeded on 0.001% poly-L-lysine-coated (Sigma) glass coverslips onto 24-well plates (Nunc) at a density of 2.5 × 10^5^ cells/cm^2^. The culture medium (500 μl/well) was exchanged after 24 h and cultures allowed to mature *in vitro* for 7–10 days before transfection.

### Transfection of siRNA in Murine Primary Microglia Using the OZ Bioscience Magnetic Plate Technology

We followed the manufacturer’s recommendations with a few modifications (Supplementary Figure S1). The amounts used here refer to a 24-well plate format. In one microcentrifuge tube we added 0.6 μl of siRNA (stock concentration is 20 μM) and we mixed by vortexing with 100 μl of DMEM without serum or antibiotics. The contents of this tube were added to a new tube with 0.4 μl of Glial-Mag where the contents were mixed gently by pipetting up and down 4–5 times. The mix was incubated at room temperature for 20 min. During this incubation period, we removed 100 μl of media per well from the plates where primary cells were seeded using the previously described complete media to assure a final volume of 400 μl of media per well. The content of the microcentrifuge tube was then added drop by drop to each well together with 5 μl of Glial boost 100×, the second component in the Glial-Mag kit. We placed the culture plate inside of the cell incubator on top the magnetic plate provided by the manufacturer for 30 min. Afterwards, we removed the magnetic plate and left the cells in the incubator for 3 h at 37°C. We then exchanged the media for conditioned media saved from microglia co-cultured with astrocytes (ratio 1:3 fresh media: old media; in the case of pure microglial cultures) or complete media as described above (in the case of cerebellar granule cell (CGC) cultures) and waited for 48 h before we performed the experiments. A description of the different steps for transfection are included in Supplementary Figure S1. The different siRNAs used for this study were siGLO Green (positive control for delivery #D-001630-01) ON-TARGETplus Non-targeting Pool (#D-001810-10), ON-TARGETplus Mouse Cd33 siRNA (#L-047462-01), ON-TARGETplus Mouse Trem2 siRNA (#L-040918-01) and ON-TARGETplus Rat Trem2 siRNA (#L-082332-02). The sequences of the different siRNAs are provided in Table 1.

### Quantification of Cell Numbers in CGCs and Pure Microglia Cultures and Analysis of the Percentage of Cells Transfected With siGLO

Following the same protocol of transfection as with primary cortical microglia cells, 3 h and 30 min after transfection CGC cultures were washed once in PBS and fixed with 4% paraformaldehyde (PFA, in PBS, pH 7.4), and washed three more times with PBS. Then, fixed cultures were stained with the nuclear dye Hoechst 33342 (5 μg/mL) and 568-tagged isolectin-B4 (1 μg/mL) was used to identify microglia. Healthy and apoptotic (chromatin-condensed) neurons were recognized by their distinct nuclear morphology, whereas bigger and more diffuse nuclei stained by Hoechst 33342 were scored as astrocytes. siGLO was used to assess the % of cells that were transfected. The % of siGLO positive cells was measured as 100× (number of microglia or astrocytes or neurons that contain siGLO/total number of microglia or astrocytes or neurons).

Similarly, both CGC cultures and pure microglia cultures were kept inside the incubator 48 h after transfection. Then, cells were stained with Hoechst 33342, and 568-tagged isolectin-B4, as previously indicated. In all the experiments, four microscopic fields/well (between 150 and 250 neurones per field) in 1–2 wells/condition were quantified for a single experiment. Total cell densities and % of siGLO positive cells were evaluated using a Leica DMI6000 CS microscope and cell densities were quantified using the cell counter plugin on ImageJ software.

### RT-qPCR Analysis

Total RNA was extracted using the QIAzol Reagent (QIAGEN) following the manufacturer’s instructions. Using the RevertAid First Strand cDNA Synthesis Kit (Thermo Scientific, UK), 1 μg of the total RNA was transformed into cDNA. RT-qPCR was performed using the MESA BLUE SYBR^®^ Assay (Eurogentec, UK). Results were calculated using delta Ct method and represented as absolute values with arbitrary units. β-actin was used as the housekeeping gene. The primer sequences are provided in Table 2.

### Preparation of Labeled Neuronal Debris

To generate stained neuronal debris, mouse and rat CGCs originated from P3 to P5 were used (which are 90% neurons). Cells were washed twice with PBS and after the second wash, 100 μl of PBS was left in the well (24-well plate format) into which the cells were scraped. Then the cells were passed 5–10 times through a 27G syringe needle. After that, we incubated the neuronal debris with tetramethylrhodamine (TAMRA; 50 μM) for 20 min at room temperature with agitation and protection from light. Excess of TAMRA was removed using a 5 kDa spin column (spin columns were previously left in the hood for 15 min under UV light). Columns were washed twice with PBS (200 μl) at 8,050 g for 10 min before stained debris (200 μl) with 200 μl of PBS were added and centrifuged at 8,050 *g* for 10 min twice. After each centrifugation, PBS containing free TAMRA was discarded and replaced with 200 μl of fresh PBS. After the second wash stained debris from the column was collected and protein concentration was measured using Pierce™ BCA protein assay kit (ThermoFisher Scientific).

### Analysis of Phagocytosis by Flow Cytometry

Microglial phagocytic activity was assessed by evaluating the uptake rate of TAMRA-stained neuronal debris as previously shown (Hornik et al., 2016) with slight modifications. Briefly, pure microglia cultures were incubated with TAMRA-stained neuronal debris (60 μg/ml) for 1 h inside the cell incubator. The culture media was then removed and 100 μl of 1× trypsin (0.1%) was added to each well and incubated for 5 min in the incubator. To stop the reaction, 500 μl of culture medium was added. Cells were collected and transferred to a microcentrifuge tube and centrifuged for 5 min at 150 *g* at room temperature. The supernatant was removed, and cells were fixed with 50 μl of 4% PFA (in PBS) for 15 min at room temperature using agitation. After centrifugation at 150 *g* for 5 min at room temperature, PFA was removed and cells were resuspended in 100 μl of cold PBS. Samples were kept on ice before the analysis by flow cytometry. The FL3 (excitation 640 nm, emission >670 nm; red; TAMRA in neuronal debris) fluorescence of the cells was measured using a BD Accuri C6 flow cytometer (BD Biosciences, San Jose, CA, USA). Cells not treated with TAMRA-stained neuronal debris were used to set the flow cytometry gates. The average of the TAMRA-positive fluorescence in cells treated with non-targeting siRNA was determined and compared to the fluorescence of cells treated with the TREM2 siRNA. Values were normalized to the fluorescence of cells treated with non-targeting siRNA and TAMRA-stained neuronal debris. Flow cytometry analysis was performed using BD Accuri C6 software.

### Quantification of IL-6 Release Into the Media

Supernatants of the different cell culture treatments were collected and fast freeze on dry ice and stored at −80°C until further use. We analyzed the content of IL-6 in the media by using the Mouse IL-6 ELISA MAX™ Deluxe Sets (Biolegend Cat. No. 431304) following manufacturer’s instructions.

### Statistical Analysis

Data normality and homogeneity of variances were analyzed using the Shapiro-Wilk and the Levene’s tests, respectively. All data were normally distributed. Results were tested for statistical significance using one-way ANOVA analysis of the variance with a Tukey’s multiple comparisons *post hoc* analysis using the Statgraphics or GraphPad Prism software. For those results that do not show homogeneity of variance, an unequal variance two-tailed *t* test, (Welch’s *t* test) was used. *P* < 0.05 was considered statistically significant. The number of independent experiments and statistical test used is described in the relevant figure legends. Each independent experiment represents a separate mouse or rat litter.

## Results

### Delivery of siRNA and Efficacy of Knockdown in Primary Microglia Cells

**Figure 1:**
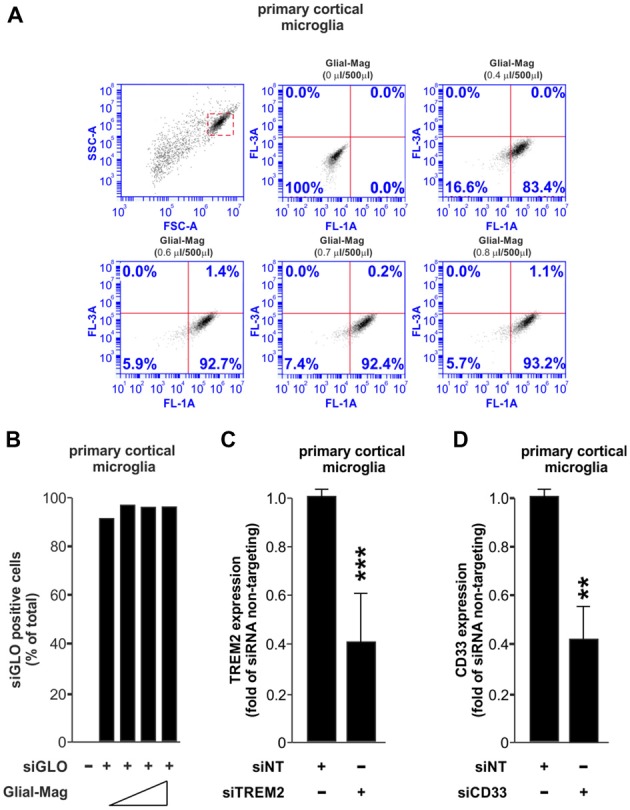
Small interfering RNA (siRNA) delivery and efficiency transfection analysis using the Glial-Mag technology. **(A,B)** Assessment of the delivery of siGLO reagent through flow cytometry represented by dot plots and histogram into mouse primary cortical microglia cells using increasing amounts of Glial-Mag. Analysis of triggering receptor expressed in myeloid cells 2 (TREM2; **C**) and CD33 **(D)** gene expression after transfection with specific siRNA measured by room-temperature (RT)-qPCR. Results are presented as mean **(B–D)** ± SD **(B,C)**. Data are from one **(A,B)** or five **(C)** or three **(D)** independent experiments. **P < 0.01 and ***P < 0.001. All analyses were performed using two-tailed Welch’s *t*-test.

Our first experiment was to assess the amount of Glial-Mag necessary to obtain a satisfactory delivery rate of a fluorescently-labeled RNA oligonucleotide (siGLO Green 6-FAM, henceforth referred as siGLO) into the primary microglial cells. For that reason, in a 24-well plate format we added different volumes of Glial-Mag together with a fixed dose of siGLO (same dose as used with siRNA TREM2 or siCD33) to the primary microglial cells. Three hours and 30 min after treatment with siGLO, cells were collected to analyze the degree of delivery of siGLO by flow cytometry (Figures 1A,B). We observed a similar degree of delivery at all doses tested (volume per well of Glial-Mag added: 0.4 μl/500 μl, 0.6 μl/500 μl, 0.7 μl/500 μl and 0.8 μl/500 μl), resulting in 83%–93% of the microglia containing siGLO at this time point (Figures 1A,B). Subsequently, all the experiments were conducted with the lowest dose of Glial-Mag (0.4 μl/500 μl per well).

We then used specific siRNAs against either TREM2 or CD33, combined with the magnetic nanoparticles of the Glial-Mag medium, and incubated with the microglia on top of a magnet to enable penetration of the siRNAs, as described in the “Materials and Methods” section and Supplementary Figure S1. We then analyzed the efficacy of knockdown by siTREM2 and siCD33 at the mRNA level measured by RT-qPCR in mouse primary cortical microglia. In both cases, we observed that the mRNA level was decreased by about 60%, 48 h after siRNA transfection (Figures 1C,D).

### Effect of Protocol on Microglial Survival and Inflammatory Response

**Figure 2:**
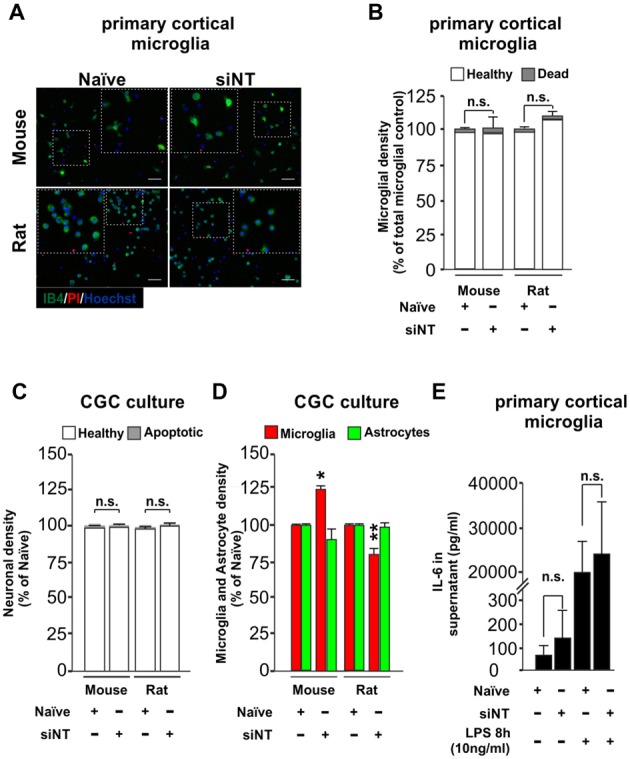
Effect of Glial-Mag technology over cell survival and glia proliferation upon 48 h transfection in primary cortical microglial and cerebellar granule cells (CGCs) primary cultures in wild type mouse and rats. **(A)** Representative images and insets showing microglia (IB4, green), PI (as a necrotic marker, red) and nuclei (Hoechst, blue) staining in cortical primary cultures from wild type mouse and rat transfected with or without siRNA non-targeting (siNT). **(B)** Quantitative analysis showing microglial density in mouse and rat cortical primary cultures transfected with or without siNT. **(C,D)** Quantitative analysis showing neuronal **(C)** and microglia and astrocyte density **(D)** in mouse and rat CGC primary cultures transfected with or without siNT. **(E)** Analysis of IL-6 release into the media in naïve and 48 h transfected siNT treated cells ± LPS treatment (8 h; 10 ng/ml). Results are presented as mean ± SEM **(B–D)** and ± SD **(E)**. Quantitative analysis of cell numbers in **(B–D)** represent four microscopic fields (mouse) or four microscopic fields in duplicate or triplicate (rat) for each condition from three independent experiments for both mouse and rat. Data are from five (for naïve and siNT) and for three (naïve + LPS and siNT + LPS) independent experiments in **(E)**. All analyses were performed using one-way ANOVA and Tukey’s multiple comparisons *post hoc* test. n.s stands for non-significant. *P < 0.05 and **P < 0.01 compared to naïve condition. Scale bar, 50 μm.

An effective transfection protocol for primary microglia cells should aim to minimize the impact over the survival of the cells in the culture through stress, and neither should induce *per se* an inflammatory response or prime it. We wanted to know if our method of transfection meets these criteria. After 7–10 days in culture, primary cultures were transfected in the absence or presence of siRNA non-targeting (siNT) and we tested the effect of transfection over survival in primary cortical microglia cells (48 h post-transfection, Figures 2A,B) and also in neuronal/glia mixed cultures obtained from the cerebellum (3 h 30 min post-transfection Supplementary Figures S2A,B and 48 h post-transfection, Figures 2C,D). For this reason, we analyzed microglial density for the primary cortical microglia cells and also neuronal, microglia and astrocyte densities in neuronal/glial mixed cultures (Supplementary Figures S2A–D) of wild type mouse and rat. In primary cortical microglial cultures, we found no negative effect over survival in both species 48 h after transfection (Figures 2A,B). In the neuronal/glial mixed cultures we found no effect over the neuronal population (Figure 2C) but we could observe a slight change in the density of microglia (increase in mouse and a decrease in rats compared to naïve microglia) 48 h after transfection (Figure 2D).

We also transfected our primary neuronal/glia mixed cultures with siGLO for 3 h and 30 mins in mouse and rat (shown here as green dots, Supplementary Figures S2B–D) and we observed that it was efficiently incorporated not only in the cytoplasm of microglia cells (≈80% of total primary microglia were siGLO positive, as indicated by IB4 staining), but also in astrocytes (≈40%–50% of total primary astrocytes were siGLO positive, as indicated by higher nuclei morphology shown by Hoechst staining) and neurons (only ≈20% of the total neurons were siGLO positive; Supplementary Figures S2B–D). Although some green dots seemed to be located at the extracellular space, the majority of siGLO staining was located inside the different cell types, suggesting that most of the siRNA used is efficiently taken up by the cells (Supplementary Figures S2B,C). Transfection with siGLO neither induced significant changes in neuronal density nor affected the number of apoptotic neurons 3 h and 30 min after transfection (Supplementary Figure S2A). The same was true in terms of affecting microglial density. However, a small decrease in the astrocytic population was observed. These data indicate that Glial-Mag method not only efficiently transfects cortical microglia cultures but can also be used to transfect neuronal/glia mixed cultures without greatly affecting the survival of neurons and glial cells.

To test whether this transfection protocol affects the inflammatory response of microglia, we quantified the levels of IL-6, a pro-inflammatory cytokine, in the media of the transfected microglial cells. No statistical difference was observed in the release of IL-6 into the media between naïve (untreated) and siNT-treated cells (Figure 2E). Furthermore, there was also no difference in the IL-6 production of naïve and siNT cells subsequently exposed to LPS (10 ng/ml) for 8 h, indicating that this transfection method does not either inhibit or prime the inflammatory response in these cells.

### Functional Analysis of TREM2 Knockdown

**Figure 3:**
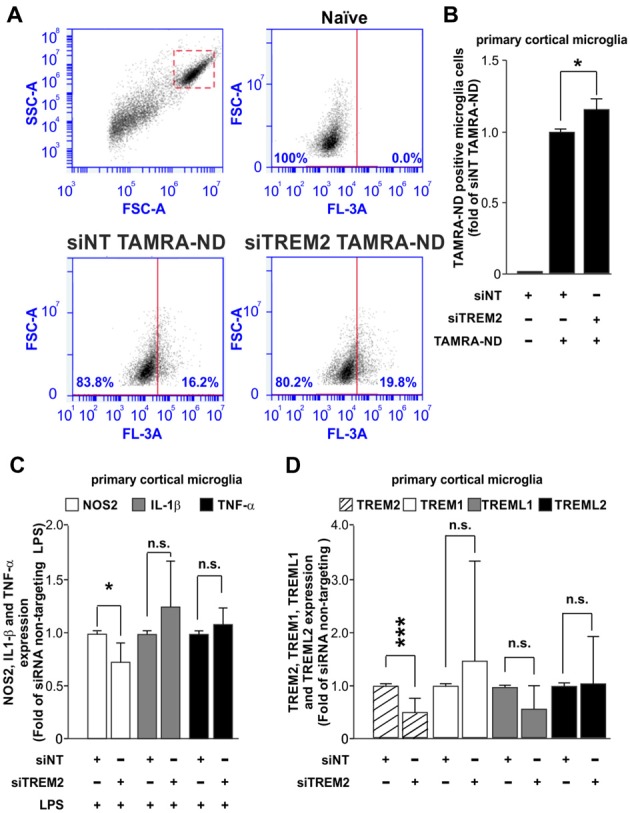
Analysis of the effect of TREM2 knockdown over the phagocytic and inflammatory responses and over the expression of other TREM family members. **(A)** Representative dot blots comparing the phagocytic response for tetramethylrhodamine (TAMRA)-labeled neuronal debris (TAMRA-ND) between siNT and siRNA TREM2 treated cells in mouse. **(B)** Quantitative analysis of phagocytosis normalized to siNT TAMRA-ND treated cells in mouse. **(C)** Effect of TREM2 knockdown over *Nos2, Il-1β* and *Tnf-α* gene expression upon LPS treatment (8 h; 10 ng/ml) in mouse. **(D)** Analysis of TREM2 knockdown on the expression of other TREM family members (TREM2, TREM1, TREML1 and TREML2) in mouse. Results are presented as mean ± SD. Data are from three **(B,C)** and five **(D)** independent experiments. All analyses were performed using two-tailed Welch’s *t* test. n.s stands for non-significant. *P < 0.05 and ***P < 0.001 and n.s stands for non-significant.

Once we had established that our system does not have an impact over the inflammatory response, we next assessed the effect of TREM2 knockdown over microglial function. Many studies have focused on the effect that mutations or loss of TREM2 have on microglial functions such as phagocytosis and inflammatory response. For this reason, we measured the effect of the siRNA of TREM2 on microglial phagocytosis of neuronal debris and the inflammatory response induced by LPS. Transfection of the microglia with siNT had little effect on phagocytosis of neuronal debris compared to non-transfected cells (naïve) treated with neuronal debris (Supplementary Figure S3A), consistent with no effect on inflammatory activation (Figure 2). In contrast, we found that knockdown of TREM2 caused a small but statistically significant increase in microglial phagocytosis of neuronal debris in mice (Figures 3A,B) and in rats (Supplementary Figures 3B–D) compared to siNT cells treated with neuronal debris cells. Regarding the inflammatory response, we found that knockdown of TREM2 reduced NOS2 expression of LPS-activated microglia in mice, while TNF-α and IL-1β expression levels remained unaltered (Figure 3C).

Some *in vivo* studies have shown that the knock down of TREM2 expression lead to an artifactual increase in the expression of TREML1 (Kang et al., 2018a), which hinders the interpretation of TREM2 knockdown effects. We assessed whether TREM2 knockdown in our study was associated with a deregulation in the expression levels of other TREM family members. We analyzed the expression of TREM1, TREML1 and TREML2 by RT-qPCR. Our results demonstrated that TREM2 knockdown using Glial-Mag method does not induce a deregulation on the levels of expression of other members of the TREM family (Figure 3D).

## Discussion

In this study, we present a simple method for successfully deliver siRNA and knockdown of gene expression in primary microglia based on binding of the siRNA to magnetic nanoparticles and magnet-induced penetration of the cells. This method does not require specific training or the use of specialized equipment for knocking down the expression of different targets. We observe with our method a high delivery rate (83%–93% of cells), even using a 3 h and 30 min incubation with the siGLO probe. Furthermore, we found that by using this method we can deliver enough siRNA to induce a 60% knockdown of TREM2 and CD33 in mice and 40% knockdown of TREM2 in rats 48 h after transfection. An additional advantage of this transfection method is the low toxicity and non-priming effect over the inflammatory response in the cultures. The lack of priming effect will provide more trustworthy data based only on the knockdown of our target gene and not due to an artifact generated by the methodology employed (as in the case discussed in Kang et al., 2018a). Our results demonstrated that the reduction in TREM2 expression observed after 48 h of transfection does not induce a deregulation in the expression levels of other TREM-related family members. These data correlate with other *in vivo* studies in which the authors did not find a deregulation of TREML1 in the original TREM2 knock out mice line (Turnbull et al., 2006) or TREM2 knock out mice generated by CRISPR/Cas9 technology (Kang et al., 2018a).

When using mixed neuronal-glial cultures, we found that this method does not distinguish between microglia, neurons and astrocytes, as siGLO entered all cell types in these cultures. This is a disadvantage if aiming to target specifically microglia, but an advantage if wanting to target all cells. However, it is important to highlight that 80% of microglial cells incorporated siGLO, while astrocytes and neurons exhibited lower rates of siGLO uptake.

In this article, we assessed the effect of knocking down TREM2 on different aspects of microglial biology. There is some controversy in the literature about what role plays TREM2 in the control of different aspects of microglia biology, including the control of the inflammatory and phagocytic responses. While several reports support the idea that TREM2 acts as anti-inflammatory protein whose total depletion or mutation (for instance R47H) promotes a proinflammatory response and reduces the phagocytic response, other reports demonstrate the opposite (Li and Zhang, 2018). These studies suggest that TREM2 has direct and indirect actions that may be contradictory. We found that partial knockdown of TREM2 expression caused a small (but statistically significant) increase in phagocytosis of neuronal debris in both mice and rats and a small decrease in LPS-induced NOS2 expression in mice. Our results may differ from other reports published by others because of the level of TREM2 depletion in the system (partial rather than total). Our results are in concordance with those for mice lacking one of the two TREM2 alleles (Ulrich et al., 2014), where there was little or no change in inflammatory markers, but a tendency for reduced NOS2 expression. Note that AD is associated with loss of function mutations in only one TREM2 gene, whereas loss-of-function in both TREM2 genes results in a different disease: Nasu-Hakola disease. Thus, in principle, partial knockdown of TREM2 may give a better idea of microglial phenotype resulting from the AD-associated mutations in TREM2. Although TREM2 is known to mediate microglial phagocytosis of neuronal debris (Kawabori et al., 2015), microglia have several other phagocytic receptors, and loss of TREM2 is known to change the expression of a wide range of microglial genes that might in principle account for our finding of a small increase in microglial phagocytosis of neuronal debris.

In conclusion, we consider that this easy method will allow many researchers to delve deeper into microglia mechanistic studies of many aspects of microglia biology using primary microglia cell cultures.

## Author Contributions

AC-J and MP performed all the experiments, except otherwise noted. AC-J and MB performed the phagocytosis analysis. MP performed and analyzed neuronal, microglial and astrocytic survival in primary pure microglial cultures and CGCs. AV contributed to conceiving and setting up the neuronal debris generation protocol, debris labeling, flow cytometry protocol and helped to prepare the primary microglia cell cultures. MB performed the RT-qPCR and helped prepare the primary pure microglia cultures. JV, GB and PS-H were involved in the study design. MB, AC-J and MP analyzed and interpreted the data. MB wrote the first draft of the manuscript. AC-J and MP contributed on writing the manuscript. All authors discussed the results and commented on or edited the manuscript.

## Conflict of Interest Statement

The authors declare that the research was conducted in the absence of any commercial or financial relationships that could be construed as a potential conflict of interest.
