# Supplementary material for: Effective Knockdown of Gene Expression in Primary Microglia With siRNA and Magnetic Nanoparticles Without Cell Death or Inflammation
Source: Front Cell Neurosci. 2018 Sep 21;12:313. doi: 10.3389/fncel.2018.00313 (PMC6161539; doi:10.3389/fncel.2018.00313)
Supplement: Supplementary file 1 [file Data_Sheet_1.PDF]

Supplementary Material

Effective knockdown of gene expression in primary microglia with siRNA and magnetic nanoparticles without cell death or inflammation

Alejandro Carrillo-Jimenez[#], Mar Puigdellívol[#], Anna Vilalta, Jose Luis Venero, Guy Charles Brown, Peter StGeorge-Hyslop and Miguel Angel Burguillos[*].

[#] These authors contributed equally to this work

*** Correspondence:** Corresponding Author: mab239@cam.ac.uk

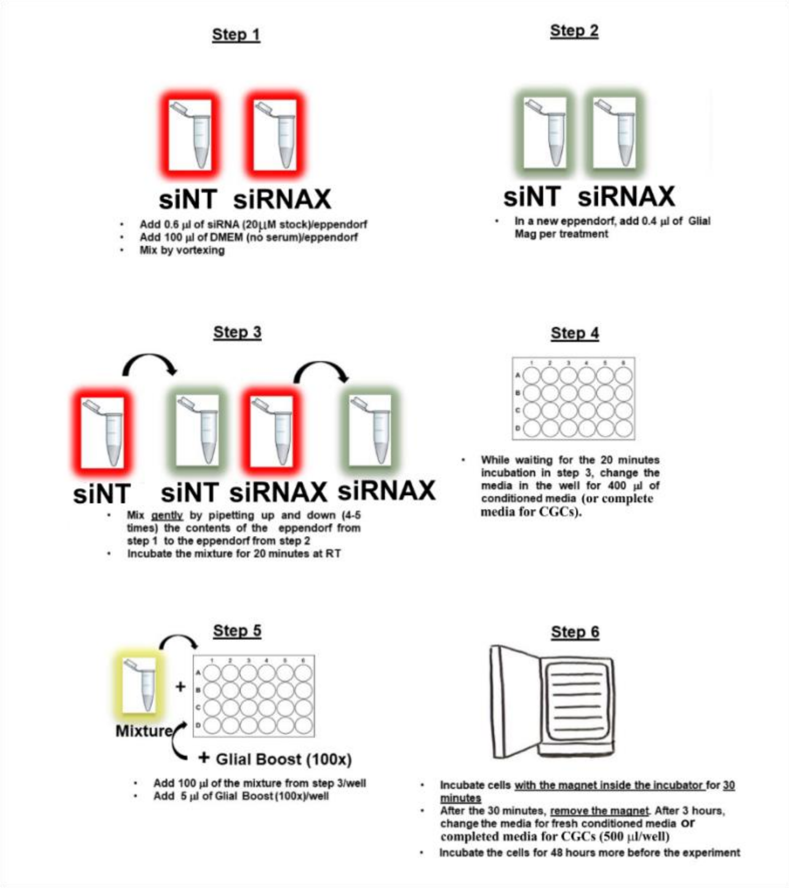

Supplementary Figure 1. Schematic representation for transfecting siRNA in primary cortical microglia cell cultures and CGCs using the Glial-Mag method**.** This protocol is for two wells of a 24-well format plate, one being for siRNA non-targeting (siNT) and the other for another target (siRNAX).
