# Supplementary material for: Effective Knockdown of Gene Expression in Primary Microglia With siRNA and Magnetic Nanoparticles Without Cell Death or Inflammation
Source: Front Cell Neurosci. 2018 Sep 21;12:313. doi: 10.3389/fncel.2018.00313 (PMC6161539; doi:10.3389/fncel.2018.00313)
Supplement: Supplementary file 2 [file Data_Sheet_2.PDF]

Supplementary Material

Effective knockdown of gene expression in primary microglia with siRNA and magnetic nanoparticles without cell death or inflammation

Alejandro Carrillo-Jimenez[#], Mar Puigdellívol[#], Anna Vilalta, Jose Luis Venero, Guy Charles Brown, Peter StGeorge-Hyslop and Miguel Angel Burguillos[*].

[#] These authors contributed equally to this work

*** Correspondence:** Corresponding Author: mab239@cam.ac.uk

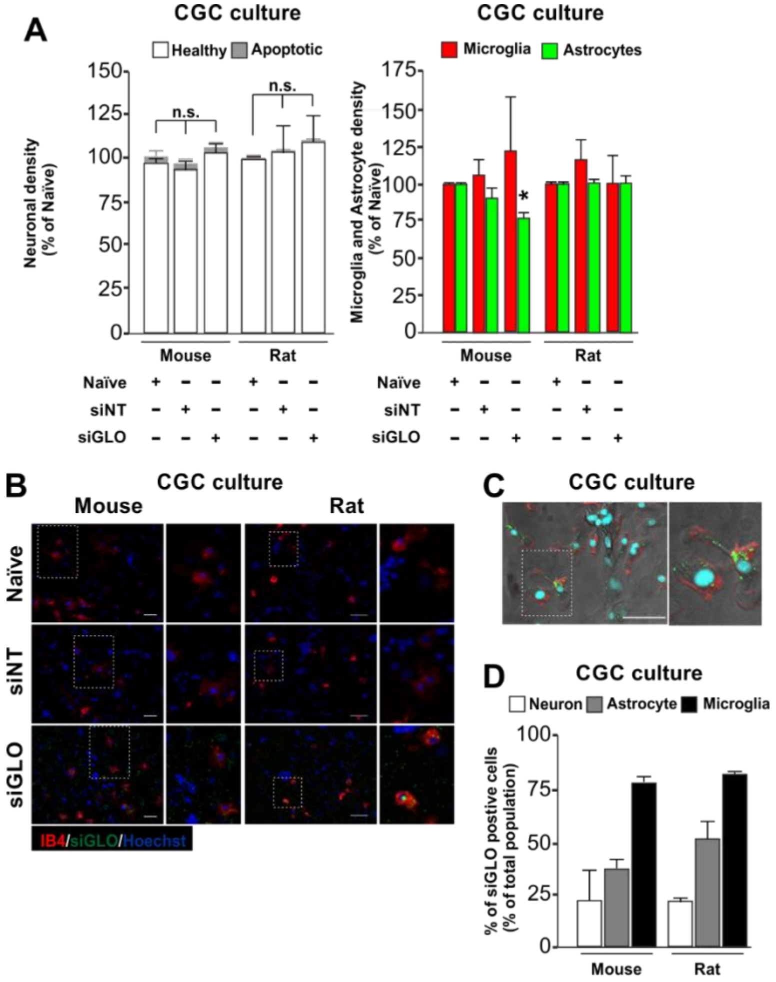

Supplementary Figure 2. Glial-Mag technology effect over cell survival or glia proliferation of CGCs wild type rat primary cultures. (A) Quantitative analysis showing neuronal cell survival (assessed by Hoechst staining), and microglia (assessed by IB4 staining) and astrocyte density (assessed by Hoechst staining), in mouse and rat wild type CGCs cultures 3h and 30mins after transfection with or without siRNA non-targeting (siNT) or siGLO. Representative images showing microglia (IB4, red), siGLO (green) and nuclei (Hoechst, blue) (B) and bright field (C) staining in CGC

primary cultures from wild type mouse and rat transfected with or without siRNA non-targeting (siNT) or siGLO. (D) Analysis of the % of siGLO positive neurons, microglia and astrocytes after 3h and 30mins transfection. Results are presented as mean ± SEM (A) and ± SD (D). Quantitative analysis of cell numbers in A and D represent four microscopic fields (mouse) or four microscopic fields in duplicate or triplicate (rat) of 4 independent experiments in mouse and 3 independent experiments in rat. All analyses were performed using One-way ANOVA and Tukey's multiple comparisons post-hoc test. n.s stands for non-significant. * $p<0.05$, compared to naïve condition. Scale bar, 50µm.
