# Supplementary material for: Effective Knockdown of Gene Expression in Primary Microglia With siRNA and Magnetic Nanoparticles Without Cell Death or Inflammation
Source: Front Cell Neurosci. 2018 Sep 21;12:313. doi: 10.3389/fncel.2018.00313 (PMC6161539; doi:10.3389/fncel.2018.00313)
Supplement: Supplementary file 3 [file Data_Sheet_3.PDF]

Supplementary Material

Effective knockdown of gene expression in primary microglia with siRNA and magnetic nanoparticles without cell death or inflammation

Alejandro Carrillo-Jimenez[#], Mar Puigdellívol[#], Anna Vilalta, Jose Luis Venero, Guy Charles Brown, Peter StGeorge-Hyslop and Miguel Angel Burguillos[*].

[#] These authors contributed equally to this work

*** Correspondence:** Corresponding Author: mab239@cam.ac.uk

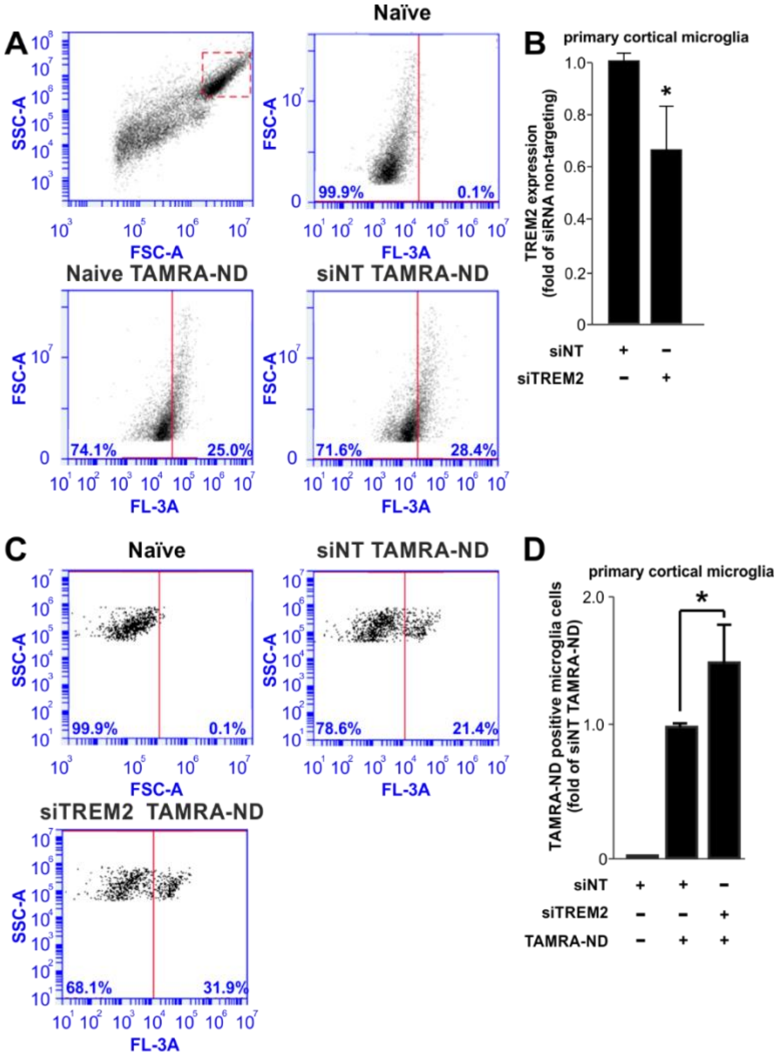

Supplementary Figure 3. Glial-Mag technology effect over phagocytosis. (A) Representative dot blots comparing the phagocytic response for TAMRA-labelled neuronal debris (TAMRA-ND) between siRNA non-targeting and naïve mouse treated cells. (B) Analysis of TREM2 gene expression after transfection with specific siRNA measured by RT-qPCR in cortical primary microglial cultures in rats. (C) Representative dot blots comparing the phagocytic response for TAMRA-labelled neuronal

debris (TAMRA-ND) between siRNA non-targeting and siRNA TREM2-treated cells in rats. (D) Quantitative analysis of phagocytosis in four independent experiments normalized to siRNA non-targeting TAMRA-ND treated cells. Results are presented as mean ± SD (B and D). Statistical analyses were performed using two-tailed Welch's t-test. *$P < 0.05$.
